# Supplementary material for: Are carbon emissions trading and green financial instruments synergistic? -Comprehensive quantitative research based on content analysis
Source: PLoS One. 2024 Mar 7;19(3):e0298601. doi: 10.1371/journal.pone.0298601 (PMC10919601; doi:10.1371/journal.pone.0298601)
Supplement: S1 Data — (ZIP) [file pone.0298601.s001.zip › Data/地级市Z_F工作报告.zip!/╔┬╬≈/╔╠┬σ╩╨/└┤╫╘╣1⁄2╓┌║┼í╛┬φ┐╦╩2╛▌═°í┐_2009.docx]

各位代表：

现在，我代表市人民政府向大会作工作报告，请予审议，并请市政协委员和其他列席人员提出意见。

一、2008年工作回顾

　 2008年是极不平凡的一年。面对经济环境复杂多变的严峻形势，全市人民在省委、省政府和市委的坚强领导下，坚持以邓小平理论和“三个代表”重要思想为指导，深入贯彻落实科学发展观，坚定信心，迎难而上，齐心协力，克难攻坚，圆满完成了市二届人大三次会议确定的各项任务，经济社会继续在科学发展、突破发展、和谐发展的道路上阔步前进。

经济健康快速增长，综合实力稳步提升。全市实现生产总值174.04亿元，增长15.2%，其中一产增加值44.58亿元，增长7.2%；二产增加值67.88亿元，增长19.8%；三产增加值61.58亿元，增长15.6%。全社会固定资产投资完成164.65亿元，增长23.6%。财政总收入完成14.25亿元，增长30.9%，其中地方财政收入完成6.82亿元，增长38.1%。城镇居民人均可支配收入10688元，农民人均纯收入2401元，分别增长20.5%和29.8%，是近几年来增幅最高的一年。

结构调整步伐加快，三次产业协调发展。积极推进农业产业结构调整和产业化经营，粮食总产达到59.46万吨，增长18.5%；新发展中药材10万亩，新建林果基地8.19万亩，新增规模养殖场户5646户，完成设施蔬菜大棚3195个、1788.8亩，无公害农产品基地发展到218万亩。劳务输出49.7万人，创收22亿元。工业经济平稳较快增长，规模以上工业增加值完成24.71亿元，增长24.7%。第三产业快速发展，全市接待游客453.23万人次，实现综合收入10.46亿元，分别增长74.3%和96.2%；社会消费品零售总额达到60.83亿元，增长24.9%；金融机构各项存款余额256.32亿元，增长24.9%，各项贷款余额97.41亿元，增长26.2%。

项目建设扎实推进，城乡面貌明显改观。60个市级重点项目全部开工建设，完成投资105亿元。产业项目建设取得突破性进展，商洛炼锌厂10万吨电解锌技改扩建、洛南九龙公司桥河钼选厂、丹凤万吨葡萄酒一期等项目建成投产，比亚迪太阳能电池、大西沟铁矿800万吨采选、镇安金岭金矿等一批重大产业项目相继开工建设。基础设施建设步伐加快，蓝商、商界和柞小高速公路建成通车，新建通乡油路374公里、通村水泥路1843公里、通达工程419公里；330千伏张鹿输变电工程及110千伏送出工程建成投运；建设户用沼气池1万口，新建基本农田8.62万亩，新增耕地1.8万亩，发展节水灌溉3.6万亩，完成营造林40万亩，新修和加固防洪河堤300公里，治理水土流失860平方公里。城镇建设力度加大，市区高速路连接线南大门、商州路东段、市区天然气管道供气和5条道路改造工程全部竣工。城市“五化”成效显著，创卫工作全面启动。各县县城及重点集镇建设快速推进，城乡面貌发生了很大变化。

改革开放不断深化，招商引资成效显著。国有资产管理体制改革取得实质进展，市级行政事业单位国有资产全部由财政部门代管。公务员、事业单位人事分配制度改革不断深化，公务员津补贴全部兑现，财政供养事业人员绩效工资预发到位。集体林权制度、种子管理体制、水务体制和农技服务体系改革取得新进展。对外合作与招商引资工作得到加强，先后与延长、比亚迪、陕西有色、中金集团等大企业、大集团建立了战略合作关系，组团参加了西洽会、农高会等大型招商活动，成功举办了“中国秦岭‘五大商药’发展论坛”和“2008•商洛核桃高端论坛”。全年招商引资到位资金38.66亿元，利用外资2633万美元，分别增长109%和274.5%。外贸进出口总额达到2673.9万美元，增长203.3%。

民生八大工程全面实施，各项社会事业协调发展。启动实施民生八大工程项目858个，完成投资17.8亿元。市政府承诺的十件实事全部完成。建设廉租房7.1万平方米、经济适用房24万平方米，住房公积金覆盖面达到75.2%。企业养老保险扩面4089人，企业离退休职工养老金按时足额发放。城镇居民医疗保险参保9.6万人，城乡低保实现应保尽保，五保对象集中供养率达到20%。新增城镇就业10887人，实现下岗失业人员再就业5438人，城镇登记失业率为3.4%。建成农村饮水工程203处，解决农村人饮困难13.08万人。实施移民搬迁1.21万人，解决了4万贫困人口温饱问题，8.1万低收入人口实现脱贫。教育事业全面发展，高考二本以上上线率居全省第一。科技创新能力明显增强，申请专利55件。城乡卫生服务体系和医疗救治体系不断完善，新型农村合作医疗参合率达到95%，名列全省第一。年度人口计划执行良好，全市人口出生率9.98‰，人口自然增长率3.01‰，出生人口政策符合率98.52%。文化、体育、广播、电视等事业蓬勃发展。节能减排工作扎实推进，单位生产总值能耗降低4.27%，化学需氧量、二氧化硫排放量分别削减2.78%和2.05%。

社会大局保持稳定，政府自身建设得到加强。政府应急管理能力显著提升，有效应对了各种突发事件。安全生产形势总体平稳，四项控制指标与上年相比均有较大幅度下降。一批信访突出问题得到有效解决，实现了“一控三降”目标。“平安商洛”创建活动不断深化，社会治安综合治理扎实有效，公众对社会治安的满意率和满意度均居全省第一。政府法制和基层民主政治建设深入推进，国防动员、军民共建和双拥工作取得新成绩。政府信息公开制度全面推行，政府系统廉政建设深入开展，部门和行业风气进一步好转。

司法、审计、统计、税务、地震、人防、气象、民族、宗教、侨务、残联、邮政、通信、档案、文史、慈善、地方志、红十字会、食品药品监管等工作都取得了新的成绩。

各位代表，过去的一年，我们经受住了多方面的困难和挑战，取得了改革开放以来最令人振奋的成就。这些成绩的取得来之不易，主要得益于省委、省政府和市委的正确领导，得益于市人大、市政协的监督支持，得益于全市人民的顽强拼搏、扎实工作。在此，我谨代表市人民政府，向奋战在全市各条战线的广大干部群众、驻商部队指战员、武警官兵和公安干警致以崇高的敬意！向关心支持政府工作的各位代表、各位委员和社会各界人士表示衷心的感谢！

在肯定成绩的同时，我们也清醒地看到，当前国际金融危机的传导效应对我市的影响十分严重，矿产品价格下跌，企业效益锐减，就业压力增大，劳务输出渠道受阻，财政减收因素较多，经济运行的不确定性增加。与此同时，我市经济社会发展中依然存在一些深层次的矛盾和问题，主要是：市场化和对外开放水平不高，非公有制经济发展不快；工业结构性矛盾突出，产业链条过短，抵御市场风险的能力较弱；农业产业化水平低，农业缺乏龙头企业带动，农民持续增收的支撑点偏少；节能减排任务艰巨，资源环境保护压力较大；一些干部的精神状态和工作作风还存在一定差距。对于这些困难和问题，我们一定要高度重视，采取更加有力的措施，切实加以解决。

二、2009年面临的形势和把握的重点

今年是商洛经济社会发展极为关键的一年，努力保持经济平稳较快发展的良好势头，既是科学应对国际金融危机的现实需要，也是确保实现“十一五”规划、加快推进突破发展的必然选择。

综观经济社会发展形势，尽管面临不少困难和压力，但是我市经济继续保持快速增长的势头不会改变，加快推进突破发展仍然面临着许多机遇和条件。一是国家扩大内需的宏观政策带来了良好机遇。中央把保增长作为宏观调控的基本取向，实施积极的财政政策和适度宽松的货币政策，出台了扩大内需的十项重要措施和一系列有利于我市发展的政策措施，这是我们加快发展的最大机遇。二是省上对陕南的支持力度进一步加大。省上支持陕南进行矿产资源规模化开采和深度加工，建设新型材料加工和制造基地，为我市加强矿产资源整合、建设现代材料工业基地提供了机遇。三是我市具有承接产业转移、加速工业化进程的有利条件。随着境内几条高速公路的建成通车，商洛真正融入了西安一小时经济圈，区位优势日益显现，这为我们接受周边城市的辐射带动，扩大对外开放，加强招商引资，承接产业转移，加快工业化和城镇化进程提供了有利条件。四是我市具有持续快速发展的良好基础和氛围。经过近几年的发展，全市蓄积了在今后一个时期持续快速发展的势能。同时，全市人民谋发展、奔富裕的愿望十分强烈，各级领导干部推动科学发展的能力不断提高，这些都是我们推动全市经济平稳较快增长的坚实基础。因此，我们完全有条件通过充分发挥主观能动性，充分调动广大干部群众的积极性，在危机中捕捉机遇，在困难中争取主动，坚定信心，因势利导，迎难而上，创造性地开展工作，把金融危机的影响降到最低程度，继续保持我市经济突破发展的良好势头。

在工作中，我们必须把握好以下几点：

一是必须更加重视发挥项目支撑作用。牢固树立“抓项目就是抓发展”的工作理念，进一步强化项目意识，积极谋划和争取一批事关全局的大项目，论证和储备一批带动作用强的新项目，推介和引进一批延伸产业链条的好项目，用项目支撑发展，用项目带动发展，用项目引领发展。

二是必须更加重视转变经济发展方式。按照稳定一产、提升二产、扩大三产的思路，推动产业结构优化升级，促进三次产业协调发展。坚持走“大集团引领、大项目支撑、集群化推动、园区化承载”的产业发展路子，引进、培育和壮大带动力强的龙头企业，延长产业链条，发展循环经济，提升经济运行的质量和效益。

三是必须更加重视把握扩大内需的政策机遇。全面落实中央保增长、扩内需、调结构的各项政策措施，以扩大投资作为推动经济增长的主要抓手，以促进消费作为扩大内需的关键环节，以调整结构作为推进产业升级的重要途径，努力实现投资、消费双拉动，形成经济平稳持续较快增长的有力支撑。

四是必须更加重视深化改革、扩大开放。坚定不移地推动思想解放和改革开放，坚持“非禁即入”原则，勇于突破制约发展的一切障碍，不断优化发展环境，全方位开展对外交流与合作，促进改革开放与加快发展的良性互动。

五是必须更加重视做好“三农”工作。始终把“三农”工作作为重中之重，以促进农业稳定发展、农民持续增收为主题，认真落实强农惠农政策，加大投入力度，推进农村改革，增强科技支撑，发展“一村一品”，提升农业产业化水平，促进农业增效、农民增收、农村发展。

六是必须更加重视改善民生、促进和谐。坚持把解决好、维护好、发展好人民群众的根本利益，作为一切工作的出发点和落脚点，着力解决好事关群众切身利益的现实问题，及时化解各类社会矛盾，维护好安全稳定、团结和谐的社会局面。

三、保持经济社会平稳较快发展

今年全市经济社会发展的基本思路是：高举中国特色社会主义伟大旗帜，深入贯彻落实科学发展观，全面贯彻党的十七大、十七届三中全会、中央经济工作会议、省委十一届三次全会和市委二届五次全会精神，坚持既定发展思路不动摇，以增加投资和刺激消费着力扩大内需，以做强特色产业促进结构调整，以强化招商引资承接产业转移，以开展节能减排带动循环经济发展，以实施民生八大工程提升公共服务水平，以创新重点领域体制机制深化改革开放，以“平安创建”活动促进社会稳定，推动经济社会平稳较快发展。

经济社会发展的主要预期目标是：生产总值完成205亿元，增长13%；全社会固定资产投资完成185亿元，增长12.4%；财政总收入完成16.55亿元，增长16%，其中地方财政收入7.92亿元，增长16%；单位生产总值能耗降低4%，化学需氧量和二氧化硫排放量分别降低3.07%和1.63%；社会消费品零售总额增长20%；城镇居民人均可支配收入12400元，增长16%，农民人均纯收入2840元，增长18%；新增城镇就业8100人，城镇登记失业率控制在4.5%以内；居民消费价格总水平涨幅5%左右；人口自然增长率控制在4.6‰以内。

实现上述发展目标，必须集中精力抓好九个方面的工作：

(一)突出重大项目建设，增强经济发展后劲。坚持把项目建设作为加快发展的第一抓手，千方百计上项目、争投入，依靠项目带动市域经济快速发展。

加快重点项目建设。今年全市安排重点项目100个，总投资574亿元，年度完成投资117亿元。柞水大西沟800万吨菱铁矿采选、比亚迪太阳能电池开发、镇安金岭金矿采选、西部鑫兴钼业深加工等50个续建项目一季度全面进入施工建设；抓紧落实建设条件，确保商洛发电厂、商州氟化工等50个新建项目上半年有80%以上的开工建设。积极配合西合、西康铁路复线建设和西商二线、榆商高速公路建设，做好商漫高速公路建设环境保障工作，积极配合做好商柞高速公路前期工作。完成330千伏张村变增容改造任务，实施中心城市、黄龙等110千伏输变电建设和改造工程。同时，扎实抓好农村基础设施、城市建设、社会事业等项目。

切实强化项目融资。瞄准国家投资方向和重点，策划、论证、包装一批优势项目，积极争取中省扶持，吸引民间和外来投资。盘活城市存量资产，充分发挥城投公司、担保公司的作用，加强与国家开发银行及各商业银行的合作，制定激励政策，鼓励引导金融机构支持重大项目建设。建立顺畅的项目资金拨付机制，确保各类项目资金及时足额到位。

重视项目前期工作。今年市上筹措2000万元，每县区拿出500万元，启动412个预计总投资1482亿元的重大项目前期工作，重点抓好柞水大西沟百万吨特钢、商丹循环工业经济园区规划项目、商南丹江梯级电站等30个重大项目前期工作，做到开工一批、论证一批、策划一批、储备一批，确保项目有续衔接、投资长期稳定快速增长。

(二)提升二产发展层次，做大做强工业经济。进一步优化工业结构，着力提高工业经济的运行质量和效益，全年规模以上工业总产值增长26%以上，增加值增长15%以上。

打造现代材料工业基地。积极申报矿产资源勘查项目，多方争取资金投入，加大勘查工作力度，确保矿业经济可持续发展。按照循环经济发展理念，制定出台现代材料工业基地建设规划。依靠大企业、大集团，加强矿产资源整合，促进矿产资源规模开发、综合利用，重点推进钼、钒、硅、铁、金、萤石等矿产深加工和新材料项目建设，不断延长产业链条，推动矿产业由采掘向深加工转变，努力建设现代材料工业基地。

大力支持工业企业发展。认真落实支持工业经济发展的政策措施，采取一企一策的办法，确保停产半停产企业一季度全部复产。为缓解工业发展资金瓶颈制约，市上设立1000万元企业发展专项资金，对推进产品深加工、实现资源循环利用和新产品开发的重点企业进行扶持。完善中小企业信贷担保体系，加强银企合作，解决融资难题。引导企业走集团化道路，支持企业上市融资。全面清理行政性事业收费，切实减轻企业负担。完善工业经济运行监测机制，强化骨干企业运行情况监测和分析，提高企业经营管理水平。

加快推进科技成果转化。认真研究制定科技带动的政策措施，进一步提高科技对经济增长的贡献率。积极申报科技创新项目、试验示范项目，力争10个以上项目列入省重大科技创新项目计划。探索建立产学研相结合的技术创新体系，加速科技成果的转化应用。加快推进企业技术改造，鼓励企业开发拥有自主知识产权的新产品、新工艺、新技术，形成一批竞争力较强的品牌、企业和产品。

强力推进工业园区建设。抓好商丹循环工业经济园区列为省级开发区的申报工作，组建园区管理机构，加快园区规划项目和基础设施建设，着手启动园区二期规划前期工作。抓住省上支持县域工业园区建设的机遇，重点推进柞水小岭工业区、洛南东部产业园、山阳中村钒业园、商南生态科技园、镇安米粮黄金工业园、柞水盘龙生态产业园、丹凤生态产业园建设，引进更多符合国家产业政策的企业入园发展，提升产业密集度，打造产业集群。

(三)落实强农惠农政策，全面推动“三农”发展。认真贯彻落实中央1号文件精神，全面推进农村改革发展工作，加快新农村建设步伐，加大农业农村投入，优化农业产业结构，发展壮大农业特色产业，促进农业稳定发展、农民持续增收。

加快农业产业化步伐。坚持以“一村一品”为抓手，加快农村经济结构调整，适当增加经济作物播种面积。大力发展干果业，完成核桃、板栗建园5万亩，良种嫁接30万亩。实施设施蔬菜工程，发展蔬菜40万亩以上。抓好道地中药材规范化基地建设，新发展中药材15万亩。因地制宜，抓好蚕桑、茶叶、烟叶等区域特色产业。加快发展畜牧业，确保全年出栏生猪120万头、家禽350万只、牛5万头、羊25万只，肉、蛋、奶产量分别达到11万吨、4万吨和1000吨。实施粮食单产提高工程，推广良种和先进耕作技术，粮食总产稳定在55万吨左右。坚持“招引”和“整合”相结合，扶持发展一批农产品加工龙头企业，提高农业产业化水平。

抓好农村基础设施建设。继续加大农村基础设施建设投入，搞好农业综合开发和重点防洪工程建设，完成商州南秦、洛南谢湾等9座病险水库除险加固任务，新修加固防洪河堤200公里，建成基本农田8.6万亩。积极发展太阳能、沼气等清洁能源，新建户用沼气池1.4万口以上。加强农村电网改造和道路建设，解决动力电到村和群众“出行难”问题。完善现代气象服务体系，抓好多普勒雷达的立项和建设。

健全农村社会化服务体系。实施“万村千乡”和“双百市场”工程，改造新建日用消费品配送中心4个，发展标准化农家店300个。建立农产品质量安全监督检测体系，严格执行市场准入制度，积极发展无公害、绿色和有机食品，搞好农产品地理标识保护，认证绿色无公害农产品20个，认定产地20个。扶持发展农民专业合作社，加快培育行业协会和商会，加强农民经纪人队伍培训，完善农技推广和服务体系，提高农业生产的组织化程度。

大力发展劳务产业。抓好农民工技能培训，完善劳务输出网络，加强中介服务和就业指导，扩大有组织的劳务输出，着力打造商洛劳务品牌，提高外出务工者收入水平。全年完成劳务输出45万人以上，创收20亿元以上。支持鼓励有一技之长的农民工在当地发展“一村一品”，依托产业就地就近转移农民。加强法律援助和维权保障，规范劳动力市场秩序，维护务工者的合法权益。

加大扶贫开发工作力度。按照“突出重点、整合资源、资金捆绑”的原则，大力推进扶贫重点村建设，新启动建设扶贫重点村112个。实施“百村贫困人口搬迁”工程和“百村互助资金”项目，投放产业扶贫贷款1.5亿元，解决10万贫困人口的温饱和脱贫问题，把返贫率控制在5%以下。

(四)着力开发生态旅游，大力发展第三产业。加大生态旅游开发力度，带动第三产业发展，努力培育新的经济增长点。

做精做优生态旅游业。以打造秦岭最佳生态旅游地为目标，积极引进战略伙伴，合作开发旅游景点，突出抓好金丝峡4A景区二期工程以及木王、牛背梁、天竺山森林公园建设，确保木王、牛背梁景区年内对外营运，努力打造金丝峡——天竺山——木王——牛背梁生态旅游圈。注重打造人文景点，力争把“闯王”包装成旅游品牌，把凤凰古镇、漫川古镇、龙驹古镇、云盖寺古镇建成有影响力的旅游景点。培育发展休闲性、参与性、娱乐性强的旅游项目，积极开发地域土特产品、民俗文化产品、民间工艺品和旅游纪念系列产品，推进特色演艺进景区，提升生态旅游整体水平。加大旅游宣传推介力度，开拓国内旅游市场，确保全年接待游客530万人次以上，实现旅游综合收入15亿元以上。

积极发展现代服务业。制定加快第三产业发展规划和实施意见，改造提升传统服务业，培育发展现代服务业。积极创造条件引进国内外金融机构来商洛设立分支机构，健全完善金融服务体系。大力培育保险市场，推进保险市场多元化。加快推进通信、网络、传媒等信息服务业发展，提高信息化服务水平。充分发挥文化资源优势，开发文化精品，满足不同消费群体的精神文化需求。支持发展经纪、策划、创意、咨询、公证、广告、设计、评估等中介服务业，大力开发社区商业、物业、家政等服务性消费，积极培育休闲、健身、保健、养老等新的消费热点。

加强城乡消费设施建设。加快建设以餐饮、住宿、娱乐为主的服务设施，建成一批星级酒店，搞好农家乐建设和星级评定，建立娱乐业投入的激励机制，建设一批中高档娱乐消费场所。抓好商洛农特产品物流中心、商南火车站物流配送中心、山阳西关商贸物流中心等现代物流市场建设，培育一批具有较强竞争力的大型流通企业。大力发展连锁经营、物流配送和特许经营，积极发展仓储、中转等货运代理服务。支持农产品批发市场、农资物流配送网点和农村“新网工程”建设，结合“家电下乡”活动，3年内在每县区发展一个家电连锁配送企业。

(五)不断深化改革开放，扩大招商引资规模。加快推进重点领域和关键环节的改革，努力在促进非公有制经济发展和扩大招商引资规模等方面取得新突破。

进一步深化各项改革。认真贯彻中央关于推进农村改革发展若干重大问题的决定，稳定和完善农村基本经营制度，引导农民以多种形式流转土地承包经营权，发展适度规模经营。对经批准占用农村集体土地建设的非公益性项目，允许农民通过多种形式参与经营，增加资本收入。抓紧做好农村服务体系和种子经营管理体制改革，全面推进集体林权制度改革。继续通过兼并、相互参股等多种形式，加快推进企业重组，鼓励市内企业与省内外大企业进行全方位合作。引导企业深化内部改革，加强管理，改善经营，提高抗风险能力。稳妥推进国有企业依法破产工作，有效盘活企业资源。创新财政资金投入方式，逐步建立以贴息、担保、参股和风险投资为主的投入机制，逐步形成财政资金围绕信贷资金安排、信贷资金围绕优势项目投放的运行模式。整合财政事业性投入，打破部门和条块分割，集中力量办大事。深入推进事业单位人事分配制度改革，率先在科研机构和演艺团体进行试点。

着力抓好招商引资。坚持把承接产业转移作为今年对外开放的重点，加大在“珠三角”、“长三角”招商引资力度，努力引进大企业、大项目落户商洛。加快研究融入西安一小时经济圈的具体措施，主动承接西安对我市的辐射带动作用。进一步加强招商引资平台建设，采取以商招商、网上招商、叩门招商等多种形式，有针对性地开展招商引资活动。积极组团参加西洽会和农高会，重点办好商洛首届核桃节和全国核桃大会，力争完成招商引资到位资金50亿元以上。进一步优化进出口结构，努力保持对外贸易快速增长。

大力发展非公有制经济。抓住平台建设、示范培植和服务引导三个重点，促进非公有制企业向规模化、集群化、科技型方向转变，全年新增私营企业220户，新增个体经营户1900户，非公有制经济增加值占全市生产总值的比重达到47%以上。加快刘湾项目区中小企业创业园的规划和建设，制定扶持创业行动计划，建立创业引导基金，完善创业服务体系，强化创业能力培训，激发各类人才的创造活力和创业激情，营造全民创业的良好环境。

(六)强化城市建设管理，打造宜居宜业城市。坚持高起点规划，高标准建设，高效能管理，不断增强城市的承载力、聚集力和辐射力。

加强城镇规划和建设。制定出台商洛市规划管理办法，围绕建设西安第二生活区，修订完善中心城市总体规划，搞好各县县城发展规划和村镇建设规划。抓紧实施南大门二期、丹南新区道路、金凤山公园二期、西街和莲湖东侧旧城改造等一批重点工程，继续推进城市“五化”。扎实抓好各县县城和小城镇、小集镇建设，积极引导农民向小城镇转移，进一步扩大建制镇规模。

提高城市经营管理水平。深入推进中心城市创卫工作，营造安全有序、整洁美观、文明和谐的城市环境。积极探索执法与服务相结合的城市管理模式。扎实推进市、县两级联创活动，全面开展商南、丹凤、山阳三县省级卫生县城创建，以及洛南、镇安、商南三县省级园林县城创建工作。抓好城市资源的开发和利用，多渠道筹集城市建设资金。

加快推进房地产开发。按照“统一规划、合理布局、综合开发、配套建设”的原则，加大经济适用住房、廉租住房、商品房建设力度，全年实施房地产开发面积51万平方米，完成投资5亿元。认真落实扶持房地产业发展的信贷政策，切实把首付按揭贷款降低到国家规定标准。加快住房制度改革步伐，规范住房二级市场交易行为。

(七)扎实抓好节能减排，提高经济社会发展水平。以更加自觉的态度、更为有力的措施，加强生态建设，推进节能减排，保护资源环境。

强化节能减排工程措施。加快实施节能工程，抓好20户重点企业的节能降耗工作。综合治理洛南黄龙钼矿区、柞水小岭铁矿区、山阳中村钒矿区等重点矿区污染，有效控制主要污染物排放。加快市污水处理厂建设进度，启动半数以上县的污水处理厂建设。实施村镇环境综合整治工程，严格控制农村污染。抓住省上实施“气化陕西”工程的机遇，做好通往我市天然气管道建设的前期工作，争取早日开工建设。 节约集约用水用地。大力推进农业节水技术改造工程，新增节水灌溉面积3万亩。加大城市供水管网改造力度，积极推进企业循环用水，促进公共建筑、小区和住宅节约用水。加强耕地保护，继续实施城乡建设用地存量挖潜改造工程，加大农村“空心村”改造和城市闲置土地清理力度，完成土地开发整理4万亩、低产田改造3万亩。

搞好生态环境建设。积极推进退耕还林、天然林保护等林业重点工程建设，完成工程造林40万亩。加强重点区域造林绿化，突出抓好中心城市城周绿化工程。大力实施小流域综合治理、南水北调中线水源保护和水土保持等项目，搞好矿山生态环境治理，治理水土流失1000平方公里。

推进循环经济发展。以贯彻实施《循环经济法》为契机，抓紧研究制定政策措施，积极探索区域循环经济发展模式，形成资源利用好、环境污染少、综合效益高的产业链。策划实施若干个尾矿综合利用项目，加快发展新型环保产业，鼓励企业对废物进行充分利用。积极推广果、畜、沼一体化生产，实现资源高效利用和循环使用。

(八)大力发展社会事业，促进社会文明进步。重视各项社会事业协调发展，促进公共服务均等化，提高全市社会公共服务水平。

坚持教育事业优先发展。继续加强基础教育，切实解决农民工子女上学难问题，推进义务教育均衡发展。加快“双高普九”创建步伐，抓好商洛中学、商南中学、镇安中学省级示范性高级中学创建工作。大力发展职业教育，改革办学体制机制，加快职业技术学院新校区建设。鼓励和规范社会力量办学，引导民办教育健康发展。进一步健全贫困家庭子女救助体系，让所有的孩子都能上得起学。加强师资队伍建设，积极推进新课改，全面实施素质教育，不断提高教育质量。

加快发展卫生计生事业。继续抓好城乡医疗卫生机构建设，加快市中心医院住院大楼建设进度，完成30个乡镇卫生院标准化建设任务。完善公共卫生服务体系，做好重大传染病防治和突发公共卫生事件处置工作。巩固提高新型农村合作医疗保障水平，抓好城镇居民医疗保险和城乡医疗救助。做好妇幼保健工作，实现农村妇女免费住院分娩补助政策全覆盖。切实抓好食品药品质量监管，保障人民群众饮食用药安全。认真落实各项奖励扶助政策，加强流动人口计划生育管理与服务，综合治理出生人口性别比偏高问题，稳定低生育水平，确保全市人口出生率控制在11.5‰以内，出生人口政策符合率保持在95%以上。 大力发展文化体育事业。加强基层公共文化基础设施建设，完成840个20户以上自然村广播电视“村村通”和七县区广播电视节目无线覆盖工程。积极推进文化体制改革，培育发展文化产业，构建公共文化服务体系，推动文化事业健康发展。加强城乡体育设施建设，完成市体育馆主体工程、体育场一期工程和70个行政村农民体育健身工程建设任务，做好市游泳馆建设的前期工作。广泛开展全民健身活动，提高竞技体育水平。

(九)重视保障改善民生，维护社会和谐安定。坚持以人为本，高度关注民生，切实保障民生，努力改善民生，完善社会管理，推动和谐商洛建设。

增强财政金融保障能力。加大财源建设力度，积极培植骨干税源，做大市本级财政。加强税收和非税收入征管，切实做到应收尽收。进一步优化财政支出结构，严格控制一般性财政支出，尤其对会议经费、公务接待、出国出境经费等支出实行零增长。加强金融生态环境建设，激励金融机构快放贷、多放贷。加快推进农村金融服务体系建设，鼓励金融机构增加新的信贷产品，为加快发展提供有力的金融支持。 扎实抓好民生八大工程。实施好1451个民生工程项目，完成投资20.25亿元。加强与中省民生工程项目的对接，把中省下达我市扩大内需的项目，与民生八大工程建设项目有机结合起来，统筹考虑，统一安排，同步实施。完善民生八大工程财力保障机制，做到配套资金足额预算、按时拨付。实行公示制度，定期向社会公开项目进展、资金使用、项目管理情况和完成时限，确保项目“阳光”实施。

千方百计扩大就业。突出抓好职业教育和人力资源市场建设，实施“人人技能”工程，提高劳动者的就业能力。实施“零就业家庭”援助行动，确保每个家庭有一人实现就业。完善就业扶持政策，开发公益性岗位1500个，发放小额担保贷款6000万元，力争带动2000人就业。按照以全民创业促进全面就业的要求，重点支持高校毕业生、复员退伍军人、失业人员和返乡农民工创业就业。

切实强化社会保障。提高企业退休人员基本养老金，切实解决国有困难企业退休人员的医疗保险问题。积极推进农村养老保险试点，完成养老扩面1500人。继续扩大失业保险和工伤保险覆盖面，力争失业参保10.7万人、工伤参保5万人。加强社会救助体系建设，五保户供养最低限定标准提高到每人每年1856元，城乡低保补助达到全省平均水平，实现应保尽保。鼓励支持慈善事业、老龄事业和残疾人事业发展。 办好事关民生的十件实事。一是实施农村饮水安全工程。建设农村饮水工程236处，解决15万农村人口的饮水困难和饮水安全问题。二是实施农村初中校舍改造工程。投资2386万元，完成18所初中宿舍、食堂、厕所及供水供暖设施改造。三是实施农村道路建设工程。新建通乡油路300公里、通村水泥路1500公里。四是实施城市垃圾处理场建设工程。中心城市医疗垃圾处理场建成投入使用，建成六县县城生活垃圾处理场。五是实施城区公厕建设工程。规划建设市区公厕35座，当年建成20座。六是实施城区道路改造工程。完成南门路、王巷路等5条道路改造。七是启动实施莲湖公园扩建工程。逐步把莲湖公园建成集旅游观光、休闲娱乐、文化教育等多种功能为一体的生态综合性公园。八是实施住房保障工程。建设经济适用房项目7个，面积19.46万平方米；建设或收购廉租房25万平方米，对低收入住房困难家庭申请租金补贴基本做到应保尽保，新增实物配租5000户；实施农村扶贫、工赈移民搬迁和危房改造4000户，解决1.65万人的住房困难问题。九是实施就业促进工程。解决4300名下岗失业人员再就业。十是搞好社区服务体系建设。改、扩建城镇社区服务站19个，新建街道社区和县级社区服务中心4个。

全力维护社会安全稳定。加强基层应急管理，有效防控突发事件。深入开展“平安商洛”创建活动，全面实施“五五”普法规划，加强社会治安综合治理，不断增强人民群众的安全感。认真落实维护稳定“第一责任”，建立和完善社会稳定预警研判机制，畅通群众诉求渠道，加强矛盾纠纷排查化解，坚决纠正损害群众利益的行为，严防发生群体性事件，确保实现“一控三降”目标。全面落实政府对安全生产的监管责任和企业的主体责任，持续开展安全生产专项整治，坚决遏制重特大事故发生。加强地质灾害监测与防治，减少地质灾害损失。

四、进一步加强政府自身建设

大力推进管理创新，加快职能转变，进一步提高政府执行力和公信力，努力建设行为规范、公正透明、务实高效、清正廉洁的政府。

强化学习意识，坚持学以致用。大兴学习之风，注重调查研究，努力建设学习型政府。扎实开展学习实践科学发展观活动，各级政府工作人员特别是领导干部，要深刻领会和准确把握科学发展观的科学体系和丰富内涵，增强贯彻落实科学发展观的自觉性和坚定性。重视学习现代科技、市场经济、金融贸易、法律法规等知识，提高领导经济工作的能力。

强化服务意识，坚持转变职能。进一步转变政府职能，强化市场监管，提高社会管理和公共服务水平。加强对经济运行的研判，充分发挥市场在资源配置中的基础性作用，更多地依靠市场的手段解决经济发展问题。减少行政审批事项，简化办事程序，切实搞好服务。完善服务承诺、公开办事和信息发布制度，提高政府工作的透明度。

强化法治意识，坚持依法行政。自觉接受人大及其常委会的法律监督和政协的民主监督，认真办理人大代表议案建议和政协委员提案，主动加强与工会、共青团、妇联等群团组织的联系，不断改进政府工作。进一步加强审计监督，重视和支持人民群众与新闻舆论监督。加强政府法制工作，全面推进依法行政，保障公民、法人和其它组织的合法权益。

强化廉洁意识，坚持从严执政。以党性教育为重点，加强领导干部作风建设。严格执行领导干部廉洁自律各项规定，重点预防工程招投标、土地出让、医药购销、政府采购等领域的不正之风。深入开展治理商业贿赂专项活动，坚决查处损坏投资环境的人和事。健全质询、问责等制度，严肃查处各种腐败行为。严格财经纪律，厉行节约，勤俭办事，着力建设节约型政府。

强化责任意识，坚持求真务实。市政府决定今年开展“工作落实年”活动。各级政府要崇尚实干，讲求效率，狠抓落实。大力精简会议、文件，切实改进会风、文风，严格控制和规范各种检查、评比、达标活动，腾出精力谋大事、促发展，增强政府的执行力。严格实行目标责任制管理，强化目标责任考核，每一项工作都要定人员、定标准、定时限，做到有安排、有督查、有考核、有奖惩，力求取得实实在在的效果。

各位代表，时代赋予重托，奋斗铸就辉煌。尽管今年面临的任务繁重而艰巨，但我们坚信，任何困难都改变不了商洛人民创造幸福美好生活的强烈愿望，任何挑战都阻挡不了全市加快推进突破发展的坚定步伐！让我们在省委、省政府和市委的正确领导下，紧紧依靠全市243万人民，同心同德，团结拼搏，开拓进取，奋发有为，共创和谐社会建设新局面，共谱商洛科学发展新篇章！
